# Neural correlates of visual aesthetic appreciation: insights from non-invasive brain stimulation

**DOI:** 10.1007/s00221-019-05685-x

**Published:** 2019-11-25

**Authors:** Zaira Cattaneo

**Affiliations:** 1 Department of Psychology, University of Milano-Bicocca, Milan, 20126 Italy; 2 IRCCS Mondino Foundation, Pavia, Italy

**Keywords:** TMS, Neuroaesthetics, Art, Paintings, Beauty, Brain stimulation

## Abstract

During the last decade, non-invasive brain stimulation techniques have been increasingly employed in the field of neuroaesthetics research to shed light on the possible causal role of different brain regions contributing to aesthetic appreciation. Here, I review studies that have employed transcranial magnetic stimulation (TMS) and transcranial direct current stimulation (tDCS) to investigate neurocognitive mechanisms mediating *visual* aesthetic appreciation for different stimuli categories (faces, bodies, paintings). The review first considers studies that have assessed the possible *causal* contribution of cortical regions in mediating aesthetic appreciation along the visual ventral and dorsal pathways (i.e., the extrastriate body area, the motion-sensitive region V5/MT+ , the lateral occipital complex and the posterior parietal cortex). It then considers TMS and tDCS studies that have targeted premotor and motor regions, as well as other areas involved in body and facial expression processing (such as the superior temporal sulcus and the somatosensory cortex) to assess their role in aesthetic evaluation. Finally, it discusses studies that have targeted medial and dorsolateral prefrontal regions leading to significant changes in aesthetic appreciation for both biological stimuli (faces and bodies) and artworks. Possible mechanisms mediating stimulation effects on aesthetic judgments are discussed. A final section considers both methodological limitations of the reviewed studies (including levels of statistical power and the need for further replication) and the future potential for non-invasive brain stimulation to significantly contribute to the understanding of the neural bases of visual aesthetic experiences.

## Introduction

The last two decades have seen a systematic attempt to link aesthetic appreciation to its neural correlates. Neuroaesthetics is indeed a growing field of research in cognitive neuroscience which aims at studying the mechanisms and neural foundations of the aesthetic experience (for reviews, see Chatterjee and Vartanian 2014; Nadal and Skov 2015; see also Pelowski et al. 2017). The range of objects and phenomena that can be appreciated aesthetically is potentially unlimited, including natural objects, human manufacts, people and even abstract ideas (e.g., see the case of mathematical “beauty”, Johnson and Steinerberger 2019). Using mainly brain imaging techniques such as functional magnetic resonance imaging (fMRI) and magnetoencephalography (MEG), cognitive neuroscientists have investigated the neural underpinnings of preferences for simple meaningless stimuli (e.g., Jacobs et al. 2012; Jacobsen et al. 2006; Zeki and Stutters 2012), paintings (e.g., Cela-Conde et al. 2004; Vartanian and Goel 2004), sculptures (Di Dio et al. 2007, 2011), interior spaces (Vartanian et al. 2013), human faces and bodies (e.g., Di Dio et al. 2011; Martín-Loeches et al. 2014), dance (e.g., Calvo-Merino et al. 2008; Kirsch et al. 2015), music (e.g., Huang et al. 2016), and mathematical formulations (Zeki et al. 2014).

**Fig. 1:**
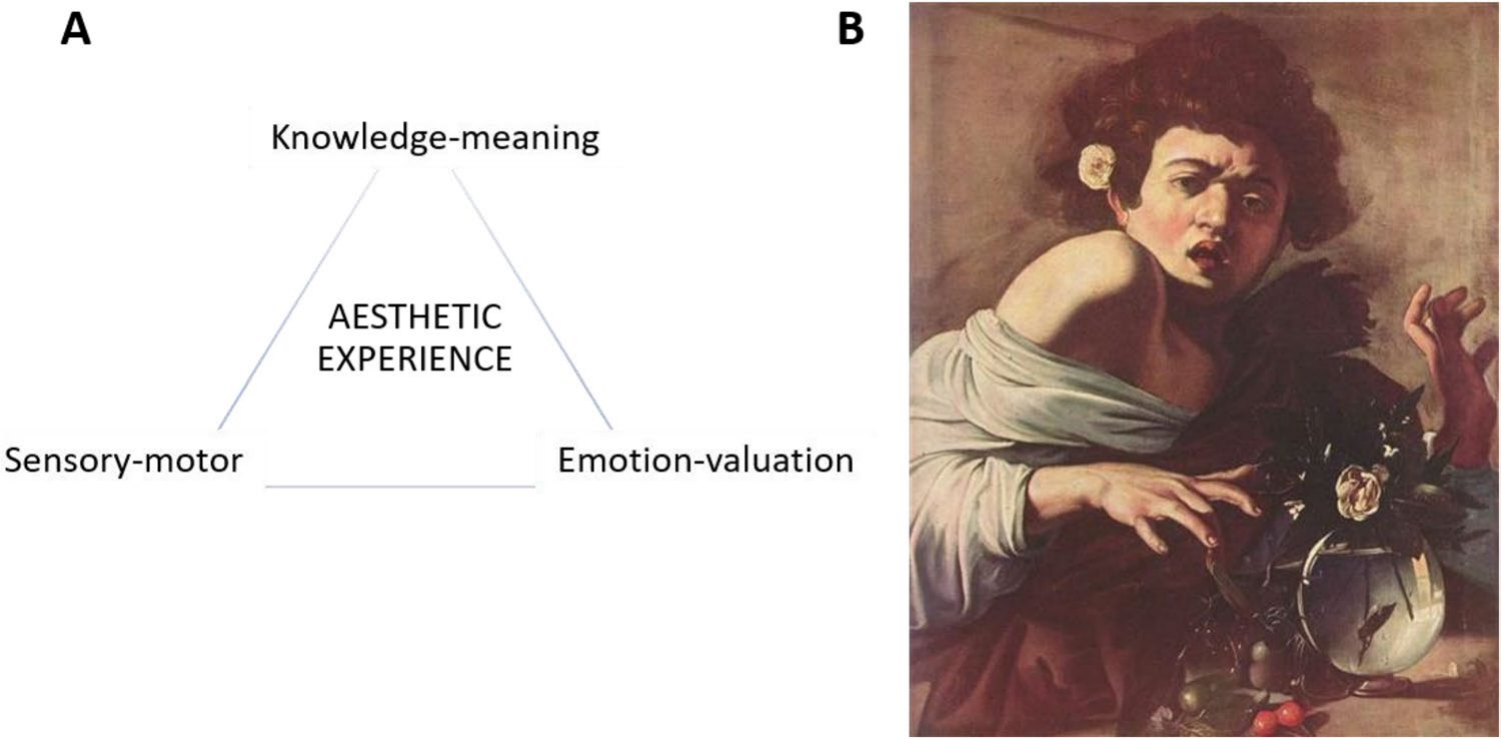
**a** The “aesthetic triad” (adapted from Chatterjee and Vartanian 2014). Aesthetic experiences can be described as emergent states arising from a flexible interaction between three main neural systems (the “aesthetic triad”, Chatterjee and Vartanian 2014): the *sensory*-*motor*, the *emotion*-*valuation*, and the *meaning*-*knowledge* systems. **b** “Boy bitten by a Lizard” (Michelangelo Merisi da Caravaggio, circa 1594-5, The National Gallery, London; reproduction free from copyright at http://www.wikiart.org). When looking at this masterpiece, the level of activation in visual regions is likely to be modulated by subjectively aesthetic preference for this painting (e.g., Vartanian and Goel 2004). The image of the boy pulling back his hand likely engages the mirror neuron system (Freedberg and Gallese 2007). This “embodied resonance” has been hypothesized to make the observers empathize with the boy’s feeling, engaging them emotionally. Moreover, engaging with art may be rewarding *per se*, thus leading to activation in reward-related brain regions (e.g., Lacey et al. 2011). Finally, contextual information (exhibited at the National Gallery) and individual art expertise may modulate neural responses throughout the brain, with activation of this extended meaning-knowledge neural system also affecting (in an exquisitely idiosyncratic way) the aesthetic experience

The notion of the “aesthetic triad” (coined by Chatterjee and Vartanian, 2014) purports that aesthetic experiences can be regarded as emergent states arising from a flexible interaction between three main neural systems: the *sensory*-*motor*, the *emotion*-*valuation*, and the *meaning*-*knowledge* systems (see Fig. 1 for an example of how these systems may interact when looking at a famous painting). Accordingly, a meta-analysis performed by Boccia et al. (2016) revealed that aesthetic judgments about visual artworks are mediated by clusters of activation in a wide bilateral network of brain regions, spanning the occipital to frontal lobes. Category-specific activations were also observed (mainly in the visual ventral stream) depending on different categories of artworks considered (Boccia et al. 2016; see also Vartanian and Skov 2014, for an earlier meta-analysis of fMRI studies investigating neural correlates of viewing paintings). Interestingly, aesthetic experiences also engage the default mode network (Belfi et al. 2019; Cela-Conde et al. 2013; Vessel et al. 2012, 2013, 2019) which is typically highly active when individuals are awake at rest or during tasks involving self-related processing, and less active during tasks requiring cognitive effort (Raichle 2015). Whilst most effort has been devoted to “localizing” the neural correlates of aesthetic appreciation, cognitive neuroscientists have also investigated brain temporal dynamics characterizing aesthetic encounters. In this context, MEG and fMRI evidence suggest that activation in visual regions, reward-related circuits, and frontal networks in response to visual stimuli may follow different temporal trajectories depending on individual liking for the images (Belfi et al. 2019; Cela-Conde et al. 2013; Vessel et al. 2012, 2013), and also on how long the stimuli are made available for visual exploration (Belfi et al. 2019).

Whilst most of neuroaesthetics literature relies on functional neuroimaging findings, in recent years non-invasive brain stimulation techniques such as transcranial magnetic stimulation (TMS) and transcranial direct current stimulation (tDCS) have also been employed to directly study the neural underpinnings of aesthetic appreciation. By interfering with and/or modulating ongoing intrinsic neural activity, TMS and tDCS are able to affect functions that rely on the targeted brain region, inducing measurable changes in behavior (see next section). TMS and tDCS are thus powerful tools providing proof of *causal* relation between brain and behavior (Filmer et al. 2014; Parkin et al. 2015). Only a few studies have employed non-invasive brain stimulation to understand the cognitive and neural processes underlying aesthetic appreciation. Yet, it is clear that this approach would be particularly fruitful for a research field in which causal neuropsychological evidence remains scarce. In fact, although studies of patients with focal brain damage have provided (and continue to provide) pivotal insights into the neural basis of behavior (Vaidya et al. 2019), research on the effects of focal brain lesions on visual aesthetic appreciation has been limited (Boccia et al. 2017; Bromberger et al. 2011; Vaidya et al. 2018).^1^

**Fig. 2:**
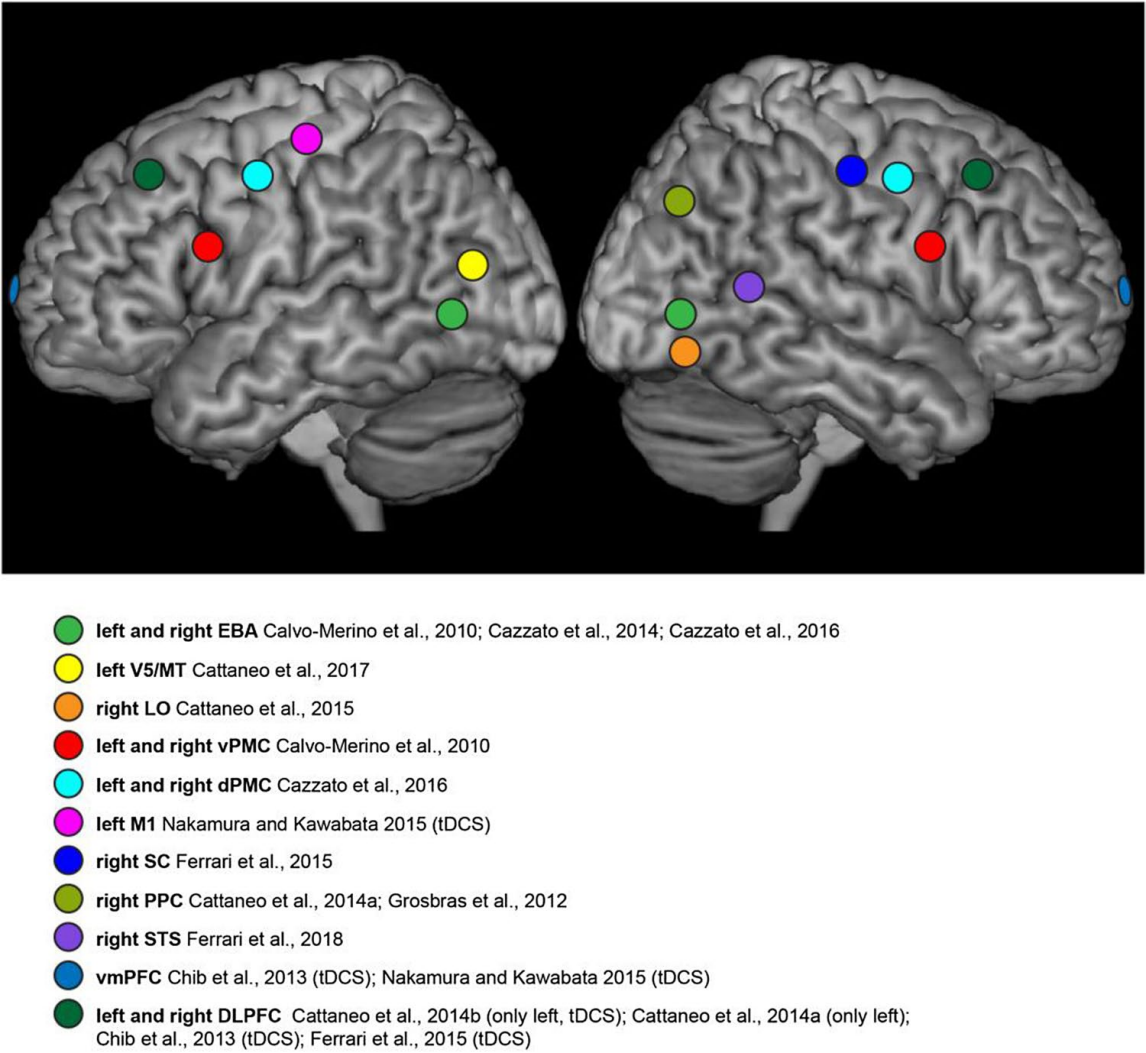
Schematic representation indicating the relative locations of different brain areas of the “neuroaesthetic system” that have been targeted using non-invasive brain stimulation (TMS; when tDCS was used instead, this is specified). *EBA* extrastriate body area, *LO* lateral occipital complex, *vPMC* ventral premotor cortex, *dPMC* dorsal premotor cortex, *SC* somatosensory cortex, *PPC* posterior parietal cortex, *STS* superior temporal sulcus, *vmPFC* ventromedial prefrontal cortex, *DLPFC* dorsolateral prefrontal cortex

This review focuses and summarizes findings from available studies (see Fig. 2 for a visual summary) that have employed TMS or tDCS to investigate the involvement of different brain regions in mediating *visual* aesthetic appreciation of paintings and of biological stimuli (i.e., faces and bodies). It is not meant to be an exhaustive review, as it only focuses on visual aesthetics and does not consider brain stimulation studies on the aesthetic appreciation of music or other non-visual stimuli.^2^ Following the model of the “aesthetic triad” (Chatterjee and Vartanian 2014), I consider how TMS and tDCS have been used to assess the contribution of nodes within the *sensory*-*motor* and *emotional*-*valuation* systems to aesthetic appreciation, and what these studies critically add to the present knowledge (which has been mainly derived by neuroimaging findings). To the best of my knowledge, there are no brain stimulation studies testing the effect of culture, context, or expertise (the *meaning*-*knowledge* system) on aesthetic appreciation, hence this remains an avenue to be explored by future research. Finally, throughout the review and in the closing section, I consider both the methodological limitations of existing studies and the potential of non-invasive brain stimulation in future investigations of the neural bases of aesthetic appreciation.

## TMS and tDCS: a brief overview of principles of functioning and of common protocols employed in cognitive neuroscience studies

Transcranial magnetic stimulation (TMS) uses a rapidly changing current within a conducting coil placed above the scalp to induce a strong, but relatively focal, magnetic field that induces a physiological response (i.e., depolarisation and/or spiking) in the underlying neural tissue (Pascual-Leone et al. 2000). TMS effects on perceptual and cognitive abilities are typically described in terms of transient and reversible “virtual lesions” caused by noise induction (Pascual-Leone et al. 2000), although, depending on the stimulation’s parameters and site, TMS may also facilitate performance (for a review see Luber and Lisanby 2014). TMS has a good spatial resolution (1–2 cm on the cortical surface) and allows to measure the contribution of an area to a specific process with a precision of few tens of milliseconds. TMS can be applied as single pulses or as trains of pulses (referred to as repetitive, rTMS); frequency of stimulation is expressed in Hz (number of pulses per second). In cognitive neuroscience paradigms, short trains (typically consisting of 2, 3 or 5 pulses at 10 or 20 Hz) are usually delivered *online*, that is concurrently with the task, on the areas of interest to temporarily interfere with stimulus encoding or with high-level processes implicated in the task. *Offline* protocols are also employed. In these paradigms, stimulation and task execution occur separately; participants are typically required to first perform the task (with no stimulation) and subsequently receive repetitive TMS (generally 1 Hz TMS for 10–25 min to suppress neural activity). Offline repetitive TMS induces changes in brain activity that last beyond the stimulation period; hence, after stimulation, task performance is re-assessed to measure any stimulation-induced effect. Control conditions (like stimulation of a control site, typically the vertex) are included in both online and offline paradigms to control for unspecific effects of stimulation. Intensity of stimulation in a study may be fixed for all participants or be individually adjusted to the cortical motor threshold (this being the preferred method when stimulating motor regions), defined as the minimal intensity of motor cortex stimulation required to elicit a reliable motor evoked potential (MEP) of minimal amplitude in the target muscle.

tDCS delivers a very weak electrical current via two surface electrodes applied to the scalp, thus altering neurons’ excitability by shifting their membrane potentials (Nitsche et al. 2008). In tDCS protocols, the current is most commonly delivered using a bipolar montage that consists in the “active” electrode (which could be the anode or the cathode, depending on the experimental design) located directly over the region of interest and the “reference” electrode located over either another cephalic or an extra-cephalic site (e.g., the shoulder). During stimulation, current flows between the electrodes (common size of each electrode is 35 cm^2^, but other sizes are possible) and, therefore, tDCS effects are not very focal. When applied to the motor cortex, anodal stimulation is known to increase neural excitability and cathodal stimulation to decrease it; these effects tend to hold also for the stimulation of other brain regions although mechanisms of anodal and cathodal stimulation may not be so straightforward outside the motor cortex (Bestmann and Walsh 2017). tDCS in cognitive experiments is typically given *offline*, that is participants perform a task before and immediately after receiving tDCS. In fact, prolonged application of tDCS can cause after-effects on the excitability of neurons and networks that outlast the stimulation. Typical intensities used in cognitive neuroscience paradigms range from 1 to 2 mA, and duration of the stimulation is commonly between 10 and 20 min. A widely used control condition consists in sham stimulation, in which the electrodes montage is the same as for real stimulation, but the current is ramped down after 30 s to 0 mA. This allows to mimic cutaneous sensations of real tDCS without affecting neural activity. *Online* tDCS protocols—in which participants perform the task whilst receiving stimulation—are also employed and seem to be particularly suited for enhancing skill acquisition (Martin et al. 2014).

## TMS evidence regarding the contribution of regions along the ventral and dorsal visual pathways to aesthetic appreciation

Several neuroimaging studies converge in suggesting that regions implied in low- and mid-visual processing of stimuli may respond differently depending on the extent to which a stimulus is preferred by the observer (e.g., Belfi et al. 2019; Calvo-Merino et al. 2008; Chatterjee et al. 2009; Lacey et al. 2011; Vartanian and Goel 2004; Yue et al. 2007). It appears that areas involved in visual perception also contribute to the computation of preferences. This hypothesis has been directly assessed using TMS.

### Extrastriate body area (EBA) and preference for bodies

The first documented study in which TMS has been used to investigate the neural bases of visual aesthetic decisions was by Calvo-Merino and colleagues (2010). These investigators applied rTMS over two regions (in both hemispheres) of the brain network implicated in body perception, the extrastriate body area (EBA, specialized in local processing of bodies according to the “dual-route model” of visual body perception, Urgesi et al. 2007) and the ventral premotor cortex (vPMC, involved in configural processing of bodies, Urgesi et al. 2007). These regions were targeted in light of a prior fMRI study finding that activity in EBA and in the vPMC was modulated by subjective liking for observed dance movements (Calvo-Merino et al. 2008). Participants were instructed to decide which of two postures of a male dancer (or control non-body stimuli derived from scrambling body ones) consecutively presented (for 100 ms each) they preferred. A train of 2 rTMS pulses (at 120% of individual resting motor threshold) was delivered, at 150 and 250 ms after the onset of the first image; pulses were timed to interfere with the cortical processing of the first image. Sham stimulation over the vertex served as control condition. TMS delivered over both left and right EBA reduced aesthetic sensitivity for body stimuli (but not for their scrambled versions) relative to ipsilateral stimulation of the vPMC and of the control site. These TMS results were the first to suggest that early perceptual analytical processing of body form in EBA *causally* contributes to the aesthetic valuation of body stimuli. According to the authors, local processing mediated by EBA may have been particularly important in driving observers’ preference for the type of stimuli presented, consisting of different postures (that only differed for leg and arm positions) of the same male dancer. Hence, in this study participants’ attention might have been driven to the detail of the body parts rather than to the global configuration.

Further support for a critical role of EBA in mediating aesthetic valuation of bodies comes from two studies by Cazzato and colleagues (Cazzato et al. 2014, 2016). In these studies, participants had to indicate on an analogue scale how much they liked a (virtual) human body. Each body image was presented for 150 ms and was immediately followed by 10 Hz five-pulse rTMS (at 110% of individual resting motor threshold), after which participants had to provide their response. In Cazzato et al. (2014), TMS was applied over both left and right EBA and over a control site (vertex). A control task was also used requiring judge the weight of the body while receiving TMS as in the aesthetic task. In Cazzato et al. (2016), TMS was delivered over the EBA and the dorsal premotor cortex (dPMC) (with half participants receiving TMS on the left hemisphere, and the other half on the right hemisphere), and over vertex as a control site. Although TMS effects were not entirely consistent across these two studies, TMS over EBA overall tended to selectively affect liking of opposite-gender bodies, resulting in increased preference. Critically, TMS over EBA did not affect body weight judgments (Cazzato et al. 2014), suggesting that the effects of EBA-TMS on the aesthetic appreciation of bodies could not be explained by changes in perceived weight. According to the authors, the enhanced preference induced by TMS over EBA may have depended on TMS interfering with the processing of local features within EBA. This in turn would have favored configural processing of bodies in premotor regions resulting in greater sensorimotor embodiment and increased aesthetic value attributed to opposite-gender bodies (Cazzato et al. 2014, 2016). Note that this effect was only induced by right EBA stimulation in female observers in Cazzato et al. (2014): there may thus be a hemispheric asymmetry in the contribution of EBA to aesthetic processing of human bodies in female observers. However, this finding was not replicated in Cazzato et al. (2016) and deserves further testing.

The studies reviewed above provide convergent evidence showing that EBA is involved in the aesthetic valuation of bodies. However, it remains unclear at this stage whether aesthetic valuation of bodies is driven more by local/analytical encoding (as suggested by Calvo-Merino et al. 2010) or by configural encoding (as suggested, at least in part, by Cazzato et al. 2014, 2016). The degree to which aesthetic appreciation relies on local and global processes may indeed depend on stimuli features (e.g., static vs. dynamic bodies, dance postures vs. ordinary dynamic postures) and on the type of decision required (e.g., evaluation of a single body, dichotomous preference for pairs of bodies), but this remains to be clarified. Moreover, the role of EBA in aesthetic appreciation of bodies should be further considered in light of consistent evidence suggesting that EBA may not just represent body parts, but it is likely to be more directly involved in motor control, providing an interface between perception and action (Zimmermann et al. 2018).

### Processing motion and content in paintings: V5/MT+ and the lateral occipital (LO) area

There is some behavioral evidence that the level of dynamism perceived in a painting may be an important factor in driving preference. In particular, Massaro et al. (2012) reported that representative paintings depicting a dynamic content were preferred to static paintings by art-naïve observers. Implied motion in static images is known to activate the motion-sensitive region V5/MT+ (e.g., Kourtzi and Kanwisher 2000; Williams and Wright 2009). Accordingly, prior fMRI evidence reported that activation responses in area V5/MT+ were correlated with perceived motion in a set of figurative (Van-Gogh) paintings (Thakral et al. 2012). Whether aesthetic preference for dynamic stimuli critically implicates V5/MT+ is not clear though. In fact, Thakral et al. (2012) did not find a correlation between activation in V5/MT+ and subjective preference for figurative paintings. In contrast, Zeki and Stutters (2012) observed that the level of V5/MT+ activation was positively correlated with aesthetic preference for simple configurations such as kinetic dot patterns.

To shed light on the possible contribution of area V5/MT+ to the aesthetic evaluation of artworks, we designed a study in which we applied TMS over V5/MT+ while art-naïve participants were asked whether they (1) perceived a number of unfamiliar abstract and figurative paintings as static or dynamic (dichotomous response required) and, (2) liked them or not (Cattaneo et al. 2017). Triple-pulse 10 Hz TMS (at a fixed intensity) was delivered over V5/MT+ or over a control site (vertex) 100 ms after onset of the painting (that remained visible till response). We found that TMS over V5/MT+ compared to vertex stimulation significantly reduced the number of paintings participants perceived as dynamic, suggesting that this area processes motion in paintings, thus strengthening prior fMRI evidence provided by Thakral et al. (2012). However, TMS over V5/MT+ also selectively affected the liking of abstract (but not figurative) paintings. This latter finding is in line with the fMRI results by Thakral et al. (2012) that did not report a correlation between V5/MT+ activation and preference for figurative paintings. In turn, the decrease in preference for abstract paintings following TMS delivered to V5/MT+ may be explained by considering that when viewing abstract art lacking a figurative content, attention of art-naïve observers is mainly captured by low-level features (i.e., motion, colors, or orientation, see Cupchik et al. 2009; Nadal 2013). Interfering with the encoding of low-level features may thus be more detrimental for the aesthetic appreciation of abstract rather than figurative artworks. The contribution of area V5/MT+ to visual aesthetic decisions may, therefore, depend on whether the paintings have a recognizable figurative content or not.

Some indirect support for this hypothesis is available from another TMS study carried out by our group (Cattaneo et al. 2015) in which we interfered with activity in the lateral occipital (LO) area. LO resides along the visual ventral stream and is specialized in object/shape encoding (Grill-Spector 2003). Furthermore, responses in this area have been correlated with subjective preference for art images (Lacey et al. 2011). In that study, we presented art-naïve participants with a number of figurative and abstract unfamiliar paintings. Triple-pulse 10 Hz TMS (at a fixed intensity) was delivered over LO or over a control site (vertex) at onset of each image. Observers had to look at each painting and decided whether they liked it or not (dichotomous response required, the painting remained visible till response). We found that TMS delivered to LO resulted in an opposite pattern compared to what we observed stimulating V5/MT+ . Specifically, TMS over LO decreased preference for figurative, but not abstract, paintings in art-naïve observers (Cattaneo et al. 2015). Since TMS over LO did not affect the sharpness/clearness of images (as we assessed in a control experiment, keeping identical TMS parameters as in the aesthetic task), we hypothesized that the selective LO-TMS effect on the preference for figurative artworks likely depended on interfering with the processing of the depicted content (Cattaneo et al. 2015). The abstract paintings we presented did not contain any recognizable objects, and, therefore, their aesthetic valuation did not involve object-recognition mechanisms mediated by LO. Given that these results are based on a single study, this hypothesis needs further empirical testing, including more appropriate control tasks to disentangle which specific mechanisms mediated by LO are important for aesthetic valuation.

### The posterior parietal cortex: spatial encoding and attentional control

Activation in bilateral sectors of the parietal cortex has also been reported during the viewing of artworks (e.g., Belfi et al. 2019; Cela-Conde et al. 2009; Cupchik et al. 2009; Di Dio et al. 2016). Parietal activity in these studies has been interpreted as reflecting the encoding of spatial relations in artworks, this possibly contributing to the understanding of the depicted scene and hence its aesthetic valuation. We investigated the possible causal contribution of the right posterior parietal cortex (PPC) in mediating aesthetic appreciation by applying TMS over this region while art-naïve participants had to decide whether they liked or not a series of abstract and figurative unfamiliar paintings (Cattaneo et al. 2014a). In this study, triple-pulse fixed intensity 10 Hz TMS was delivered 100 ms from onset of the painting over the right PPC, the left dorsolateral prefontal cortex (DLPFC) and the vertex (control site). Observers had to look at each painting and decided whether they liked it or not (dichotomous response required, the painting remained visible till response). We selectively targeted the *right* parietal cortex (corresponding to the P4 site in the standard 10–20 EEG system, Homan et al. 1987) since this side is known to be recruited more prominently than the left in visual spatial attention and representation (for a recent review, see Bartolomeo and Seidel Malkinson 2019). We found that interfering with right PPC activity resulted in decreased preference for figurative paintings, although only in participants preferring figurative over abstract art. The selective effect of parietal TMS on aesthetic preference for figurative paintings is in line with a neuroimaging study by Fairhall and Ishai (2008) that contrasted neural responses during viewing figurative paintings compared to abstract paintings or indeterminate ones (i.e., paintings implying natural forms, but without easy or immediate recognition). Fairhall and Ishai (2008) found selective patterns of activation within sectors of the parietal lobe associated with the recognition of meaningful, familiar content in figurative artworks but not for abstract and indeterminate paintings. The PPC may thus contribute to the analysis of structured meaningful scenes, and interfering with processing in this area may affect aesthetic valuation.

In interpreting the effects of parietal stimulation over aesthetic appreciation, it is important to consider that sectors of the parietal cortex are also involved in motor mapping and embodied resonance mechanisms (Fogassi and Luppino 2005) that may also be important in driving aesthetic pleasure (as hypothesized by Freedberg and Gallese 2007, see next section). Moreover, there is evidence that regions of the parietal cortex are also involved in value-based decision making, possibly through cortical interactions with more anterior regions such as the ventromedial prefrontal cortex (Larsen and O’Doherty 2014). Finally, the parietal cortex is also part of the fronto-parietal network which is important in exerting attentional control over other brain regions (Waskom et al. 2014). Thus, interfering with parietal activity may also indirectly affect activity of other more distant regions. For instance, using fMRI Grosbras et al. (2012) observed that activation within a region of the right posterior parietal cortex was negatively correlated with the dynamic emotional reactions of participants while they watched a dance video (Grosbras et al. 2012). In a follow-up experiment, they applied 15 min low-frequency rTMS to suppress activation in this region and found that this stimulation led to an enhancement in the subjective emotion rating of participants for specific moments in the dance. According to Grosbras et al. (2012), inhibiting the parieto-frontal executive control system appears to reduce “cognitive” analysis (such as judging the quality of movements, etc.…) of the observed dance, thereby freeing up resources for emotional processing and resulting in greater appreciation.

Given the multiple roles associated with parietal cortex function, different mechanisms may be responsible for the contribution of this area to aesthetic experience.

### Summary and discussion

Overall, the studies reviewed above suggest that interfering with activity in regions involved in perceptual encoding of bodies and paintings also affects their aesthetic valuation. This may admittedly not be surprising in that one would expect that interfering with encoding of low-level features of a specific stimulus would create a cascading effect on higher-order decisions about that stimulus. However, what is interesting in these findings is that they shed light on the different functional mechanisms that mediate aesthetic appreciation of different types of stimuli. For instance, the results of Calvo-Merino et al. (2010) and Cazzato et al. (2014, 2016) suggest that aesthetic appreciation of bodies may rely in part on a local perceptual analysis (in which EBA seems to be specialized, see Urgesi et al. 2007) but also on more global/configural processing depending on task requirements, stimulus features (e.g., static vs. dynamic bodies, dance-related vs. ordinary movements) and individual differences (e.g., gender of the observers). In the case of paintings, available TMS evidence suggests that different mechanisms drive aesthetic experience for abstract and figurative art (at least, when observers are art-naïve). In particular, the more the aesthetic judgment is driven by low-level sensory features (such as motion perceived in the image), the more feature-specific regions (such as V5/MT+) are likely to be implicated in contributing to the aesthetic experience (Cattaneo et al. 2017). In turn, when objects are depicted in a painting, aesthetic decisions are likely to rely more on processing in cortical regions involved in object recognition and encoding of spatial relations in structured scenes (Cattaneo et al. 2014a, 2015).

How does this TMS evidence relate to fMRI findings showing higher activation in sensory regions as a function of subjective preference? The TMS studies reviewed above show that regions along the visual ventral and dorsal pathways are involved in aesthetic valuation but they do not provide direct evidence as to why sensory regions should be more active for preferred stimuli. One possibility is that preferred stimuli somehow tap more into the type of processing in which a specific region is specialized, resulting in higher responses (as indexed by increased activation recorded by fMRI). However, this remains a speculative (and at this stage, rather circular) argument that deserves careful empirical testing. Changes in the activation of visual areas depending on subjective preference may also reflect the influence of bottom-up factors (related to stimuli’s perceptual features, such as salience) and top-down voluntary attention, with salience and attention both influencing the processing of information in sensory regions (Poltoratski et al. 2017). TMS over visual regions may thus also interfere with these mechanisms regardless of the specific judgment required (aesthetic vs. other judgments). To control for the effects of attention and salience, future studies should systematically compare TMS effects on both aesthetic and other perceptual judgments while maintaining a constant level of attention and stimulus salience.

A further valuable approach to clarify the mechanisms by which sensory regions contribute to preference would consist in measuring the effects of a specific stimulation on both behavior and neural activity. This would help bridge fMRI and brain stimulation evidence clarifying the functional significance of preference-driven responses in sensory regions. This approach has been employed in one study focusing on the midbrain’s contribution to reward computation associated to valuating face attractiveness (Chib et al. 2013; described in detail in a next section). Finally, it has been suggested that preference-driven activations in the visual cortex may also depend on the distribution of mu-opioid receptors in in this region (Biederman and Vessel 2006). Although this hypothesis deserves further investigation on its own, it is worth mentioning here that TMS and tDCS may activate the striatal dopamine and opioid systems (e.g., Chib et al. 2013; Lamusuo et al. 2017). These effects, that have been so far mainly of interest for research in neuropathic pain, are also critical when considering how TMS may be used to affect aesthetic pleasure.

## Embodiment, motor regions, and aesthetic appreciation: TMS and tDCS evidence

It has been suggested that a critical contribution to aesthetic evaluation may derive from the activation of embodied mechanisms in response to the viewed stimulus encompassing the simulation of actions, emotions and corporeal sensations in nodes associated with the mirror neuron system (Freedberg and Gallese 2007). In fact, prior fMRI studies observed activation in premotor cortices during the observation of dance movements (Calvo-Merino et al. 2005, 2006; Cross et al. 2011; see also review by Kirsch et al. 2016), sculptures and figurative paintings (e.g., Di Dio et al. 2007, 2011, 2016). As in the case of sensory cortices, activation in premotor cortices may also reflect preference for the viewed stimuli, although so far there is only preliminary evidence. Specifically, Calvo-Merino et al. (2008) found stronger activity in occipital cortex bilaterally and in the right premotor cortex when subjects viewed short dance passages that they reported liking in a later evaluation, compared to those they disliked. Importantly though, motor activations observed during art viewing may reflect other mechanisms beyond embodied “mirror” resonance, such as approach/avoidance behaviors elicited by the perceived beauty or ugliness in an artwork (Kawabata and Zeki 2004; see also Di Dio et al. 2007).

### Aesthetic preference for bodies and paintings: involvement of premotor and motor regions

In the study described in the previous section, Calvo-Merino et al. (2010) observed a tendency towards increased aesthetic sensitivity for dance body postures following bilateral application of TMS over the ventral premotor cortex (vPMC). This effect may reflect disruption of configural processing of bodies in the vPMC resulting in increased local processing by the extrastriate body area (EBA), in line with the “dual route” model of body processing according to which EBA is specialized in local processing and the vPMC in configural processing of bodies  (Urgesi et al. 2007). According to Calvo-Merino et al. (2010), the increase in local processing would have facilitated aesthetic sensitivity for dance body postures, in which local details were probably more important than the global configuration in driving preference.

Cazzato et al. (2016) also reported that TMS over the premotor cortex affected aesthetic valuation of bodies, but linked this effect to disruption of sensori-motor embodiment mechanisms. In particular, they found that interfering with the dorsal premotor cortex (dPMC) bilaterally during the aesthetic valuation of static and dynamic ordinary body postures selectively decreased liking of same-gender bodies. The authors suggested that TMS interfered with embodied mechanisms occurring in dPMC that may be important in aesthetic appreciation, embodiment being possibly triggered more by same- than opposite-gender bodies (Cazzato et al. 2016). Premotor activation may thus *causally* contribute to aesthetic appreciation of the viewed stimuli when the latter strongly drive sensorimotor embodiment. This hypothesis deserves further empirical testing, also using sculptures or paintings as stimuli, although no fMRI study has so far shown a correlation between activation in premotor cortex and liking for artworks.

The involvement of the motor cortex in aesthetic appreciation of paintings has been assessed by Nakamura and Kawabata (2015) using tDCS. This group applied tDCS to simultaneously modulate excitability of the ventromedial prefrontal cortex (vmPFC) and the left primary motor cortex (M1) while measuring the effects of stimulation on beauty and ugliness judgments on a series of abstract paintings. These sites were targeted in light of prior fMRI evidence showing that the vmPFC responds more to preferred paintings, whilst the motor cortex seems to respond more to artworks perceived as ugly, possibly reflecting motor preparation to avoid the ugly or aversive stimulus (Kawabata and Zeki 2004; Ishizu and Zeki 2011). In line with this evidence, Nakamura and Kawabata (2015) hypothesized that enhancing excitability in one of these two regions while concurrently inhibiting excitability of the other region would have affected beauty and ugliness evaluations. Participants performed beauty and ugliness rating tasks before and after the application of 2 mA tDCS (stimulation was delivered for approximately 15 min). Anodal stimulation of the left M1 (increasing excitability of this region, with the inhibitory cathode placed over the vmPFC) led to a decrease in beauty ratings, without affecting ugliness ratings. The opposite montage (cathode over the left M1 and anode over the vmPFC) did not affect beauty or ugliness judgments. The results of this study suggest that enhancing excitability of the motor cortex (combined with decreasing excitability in the vmPFC), may result in decreased perceived beauty in abstract paintings. Unfortunately, the stimulation montage used in this study does not allow us to determine whether the reported effects depended on anodal stimulation of the left M1, cathodal stimulation of the vmPFC or interaction effects between the two. Disentangling between these possibilities deserves further investigation.

### Emotional processing in portraits: targeting the somatosensory cortex and the superior temporal sulcus

In discussing embodied mechanisms contributing to aesthetic appreciation, I also briefly consider a recent experiment by Ferrari et al. (2018). In this study, we employed TMS to assess whether valuation of emotional expressivity of portraits (i.e., the degree to which a face expresses an internal/emotional state) recruits the superior temporal sulcus (STS) and the somatosensory cortex (SC). These two regions, especially in the right hemisphere, are believed to be involved in the processing of emotional expressions in real (photographed) faces (Kragel and LaBar 2016; Pitcher 2014; Pitcher et al. 2008). Two different groups of art-naïve participants were asked to indicate (by pressing a yes/no key) either whether they found a series of portraits “expressive” or whether they liked them. Each painting was individually presented and triple-pulse fixed-intensity10 Hz TMS was delivered 100 ms from onset of the painting, that remained visible till participants’ response. The portrayed faces used were only moderately expressive, since dichotomic classification of stimuli that are too polarized in terms of the feature to be evaluated would likely not be vulnerable to TMS modulation. We found that TMS over right STS significantly reduced perceived expressivity of portrayed faces but not expressivity of other paintings, suggesting that portrayed facial expressions were processed as real facial expressions by STS. In turn, TMS over the right SC did not affect expressivity judgments, possibly because the portrayed faces were not sufficiently expressive to elicit motor resonance mechanisms in this region. Neither stimulation of STS nor of SC affected liking of the paintings. While this study does not provide support for a causal contribution of the somatosensory cortex and STS to aesthetic valuation of portraits, it does fit with an interesting line of experiments assessing similarities and differences between perceiving others (such as in photographs) compared to depictions in paintings that are far removed from daily experience (Lacey et al. 2011; Lutz et al. 2013).

### Studies recording motor evoked potentials (MEP)

Other studies have targeted the primary motor cortex with single-pulse TMS to induce motor evoked potentials (MEPs) during the observation of artworks or dance (Battaglia et al. 2011; Jola and Grosbras 2013). Here, MEPs provide a means for the quantification of cortico-spinal excitability at the time of stimulation (Rothwell 1997; Bestmann and Krakauer 2015). Battaglia et al. (2011) found that observation of the gesture which Adam makes with his extended right hand to keep the sword-bearing angel at bay (as depicted by Michelangelo in his *Expulsion from Paradis,*1508–1512) increased cortico-spinal excitability significantly more than the observation of a photograph reproducing the same arm movement. This result suggests that viewing actions in paintings may enhance cortico-spinal excitability more than viewing actions performed by real agents (as depicted in static photographs at least), possibly depending on the artist’s skills in emphasizing the kinesthetic aspects of the movement. While this is a possibility, it would benefit from further testing on a larger set of images, especially considering that this finding was not replicated in the same study when participants viewed another painting by Michelangelo that also vividly depicts an arm gesture (i.e., the *Creation of Adam*). In another TMS-MEP study focusing on dance, Jola and Grosbras (2013) found that watching a live dance performance modulated MEP amplitude. Interestingly, Jola and Grosbras (2013) also reported that both visual experience and empathic ability of the observers modulated corticospinal excitability during dance observation.

Note that neither Battaglia et al. (2011) nor Jola and Grosbras (2013) measured aesthetic preference for the stimuli they presented, and it is thus not known whether cortico-spinal excitability is modulated by subjective aesthetic preference. Moreover, the results by Battaglia et al. (2011) and Jola and Grosbras (2013) cannot be interpreted as directly suggesting motor resonance mechanisms (possibly important for aesthetic appreciation, see Freedberg and Gallese 2007) triggered by dance or artworks observation. In fact, although MEPs are obtained by stimulating M1, changes in the amplitude of MEPs do not exclusively reflect the excitability of elements controlling motor output. In turn, MEPs’ changes also significantly depend on physiological processes (related for instance to decision-making or other cognitive processes) that occur outside the primary motor cortex but induce via afferent inputs into motor cortex measurable state-changes in this region (for a critical discussion on what MEPs measure, see Bestmann and Krakauer 2015). The MEP approach is certainly valuable in research of neuroaesthetics as it can provide a read-out of upstream processes related to aesthetic experience. However, these processes are not themselves necessarily related to movement production, and thus do not uniquely reflect motor-resonance or motor-preparation mechanisms.

### Summary and discussion

Overall, we have only preliminary evidence on the contribution of premotor and motor regions to visual aesthetic valuation. The studies by Calvo-Merino et al. (2010) and Cazzato et al. (2016) suggest that the premotor cortex may causally contribute to aesthetic appreciation of bodies, but the functional mechanisms mediating the observed TMS effects (i.e., disruption of configural processing of bodies in favor of local processing performed in other brain regions, interference with embodied mechanisms) remain to be clarified. Moreover, no brain stimulation study has so far assessed whether premotor regions are also involved in aesthetic valuation of artworks. Some preliminary evidence for the involvement of the primary motor cortex in aesthetic valuation of artworks can be found in the work of Nakamura and Kawabata (2015), although simultaneous stimulation of prefrontal and motor cortices as used in this former study prevents the drawing of clear conclusions about the unique contribution of the two targeted regions. Other studies (Battaglia et al. 2011; Jola and Grosbras 2013) measured MEPs as an index of motor cortex excitability while viewing paintings or dance, but they did not assess whether MEPs were affected by subjective preference for the viewed stimuli. Even if this were the case, MEPs do not uniquely reflect motor-resonance or motor-preparation mechanisms. A further TMS study (Ferrari et al. 2018) has investigated whether the STS and somatosensory cortex, regions important in processing facial expressions, play a role in aesthetic appreciation of portraits. No support for this hypothesis was found.

As a final note, it is worth mentioning that the brain stimulation studies reviewed in this section did not test art or dance experts. Prior fMRI studies have shown that observers’ expertise with dance affects the activation in premotor cortices during the observation of dance movements (Calvo-Merino et al. 2005, 2006) and learning to perform an action impacts on the neural response when watching and aesthetically valuating the same action (Kirsch et al. 2015). In future studies, the role of expertise should be investigated both by interfering with sensory and motor regions in tasks requiring explicit aesthetic judgments in expert and non-expert observers, and by measuring cortico-spinal excitability (via TMS elicited MEP) during passive viewing of the stimuli (as in Battaglia et al. 2011; Jola and Grosbras 2013).

## Reward computation and emotional engagement in aesthetic experiences: insight from stimulating prefrontal and parietal regions

Aesthetic experiences have been found to be associated with activity within different subcortical limbic regions involved in reward computation and emotional processing such as the insula, the amygdala, the ventral striatum, and the cingulate (e.g., Cupchik et al. 2009; Jacobs et al. 2012; Jacobsen et al. 2006; Vartanian and Goel 2004; Vessel et al. 2012). Moreover, fMRI studies have observed consistent activations in prefrontal regions involved in reward computation, emotional regulation and mentalizing such as the orbitofrontal (OFC) and the medial prefrontal cortex (mPFC), as well as the dorsolateral prefrontal cortex (DLPFC) when participants viewed stimuli that they considered to be beautiful (Cela-Conde et al. 2004, 2013; Ishizu and Zeki 2011; Vartanian and Goel 2004; Vessel et al. 2012). It is important to note that TMS and tDCS do not directly interfere with activity in subcortical regions in a focal manner and it is thus not possible to directly assess how subcortical regions involved in emotional processing and reward computation contribute to aesthetic appreciation. However, it is possible to indirectly modulate activity in these regions by applying stimulation to other cortical regions that are interconnected as I describe below.

### Prefrontal cortex stimulation and facial attractiveness

In an interesting study combining tDCS with fMRI, Chib et al. (2013) found that tDCS delivered to the prefrontal cortex remotely activates the interconnected midbrain (i.e., the substantia nigra and the ventral tegmental area hosting dopaminergic neurons) and causes an increase in participants’ appraisals of facial attractiveness. The study by Chib et al. (2013) was not directly focused in elucidating the neural bases of aesthetic appreciation, but rather employed a face attractiveness task as a measure of reward appraisal. In this study, participants made facial attractiveness judgements before and after receiving 15 min of 2 mA tDCS over the prefrontal cortex, and were assigned to six different stimulation conditions varying electrode location and stimulation type (i.e., active vs. sham). In the main condition, the (excitatory) anode was placed above the vmPFC (halfway between Fp1 and Fp2 on the EEG cap) and the (inhibitory) cathode was above the right DLPFC (F4 on the EEG cap). Previous studies (e.g., Meyer-Lindenberg et al. 2002; Tzschentke and Schmidt 2000) have lent support suggesting that this montage would be most effective in activating the midbrain (and hence dopamine release). In line with these expectations, Chib et al. (2013) observed that this montage not only resulted in a significant increase in attractiveness perceived in computer-generated faces, but also elicited remote functional changes within the ventral midbrain (as assessed by fMRI activation during the rating tasks). Interestingly, it was only anodal stimulation of the vmPFC with concurrent cathodal stimulation of the right DLPFC that induced behavioral changes. When the anode was kept over the vmPFC but the cathode was placed over the left DLPFC, or when the vmPFC or the right DLPFC were selectively stimulated (by placing the reference electrode over the vertex), no effects of stimulation over face attractiveness were observed. As also suggested by Chib et al. (2013), further research is needed to establish exactly which anatomical and neurochemical pathways are acted upon by the stimulation paradigm they employed.

Support for a role of the right DLPFC in possibly contributing to perceived face attractiveness can be found also in another tDCS study by Ferrari et al. (2015). In this study, we asked participants to rate their subjective preference for male and female faces prior to and after receiving 20 min of excitatory 2 mA anodal stimulation over their left DLPFC (half way between F3 and F5 on the EEG cap) or right DLPFC (between F4 and F6). The cathode was placed over the contralateral supraorbital region. We found that excitatory anodal tDCS over the right, but not the left, DLPFC enhanced perceived face attractiveness (Ferrari et al. 2015). This is consistent with prior fMRI evidence reporting modulation of activity in the right (but not the left) prefrontal cortex by face attractiveness (O’Doherty et al. 2003) and is in agreement (although not in the direction of the effects) with the selective effects reported for right (but not left) DLPFC tDCS over facial attractiveness by Chib et al. (2013). The right prefrontal cortex is functionally connected with the striatum and involved in reward computation (e.g., Koehler et al. 2013; McClure et al. 2004). By stimulating the right DLPFC we may have thus acted on fronto-striatal circuits. Moreover, anodal stimulation may have also boosted a particular type of processing important for aesthetic evaluation. More specifically, in face processing, the right DLPFC seems to be more involved in encoding of the configural/holistic aspect of faces whereas the left DLPFC is more involved with analyzing single facial features (Maurer et al. 2007; Renzi et al. 2013). If perceptual processes underlying facial-attractiveness judgments represent the face holistically (as suggested by prior studies, Abbas and Duchaine 2008), by enhancing excitability in the right DLPFC we may have boosted a configural mode of processing thus resulting in increased perceived attractiveness (Ferrari et al. 2015).

### Prefrontal cortex stimulation and aesthetic evaluation of paintings

Prior neuroimaging evidence reported selective activation in the left (but not the right) prefrontal cortex when viewing visual stimuli that participants perceived as beautiful (Cela-Conde et al. 2004) or when adopting an aesthetic (vs. pragmatic) orientation towards paintings (Cupchik et al. 2009). In light of this evidence, we assessed whether enhancing excitability of the left DLPFC via anodal tDCS would lead to an increase in subjective liking for visual stimuli (Cattaneo et al. 2014b). Art-naïve participants rated their subjective preference for a set of images (paintings, decorative sketches and photographs) prior to and after receiving 20 min of excitatory 2 mA anodal tDCS over their left DLPFC (localized as the middle point between F3 and F5 according to the 10–20 EEG system, cathode over the contralateral supraorbital region). We found that compared to a sham stimulation, excitatory stimulation of the left DLPFC induced a significant increase in liking of figurative images. In turn, tDCS neither affected liking of abstract images nor judgments of other perceptual features (i.e., colorfulness) of the abstract and figurative images, suggesting that enhancing excitability of the left DLPFC may not indiscriminately increase aesthetic appreciation.

While this review focuses on visual aesthetics, it is worth mentioning here some related tDCS work in the auditory/musical domain. In particular, Mas-Herrero et al. (2018) were able to induce significant bidirectional changes in both the pleasure from listening to music and motivation to purchase it by applying excitatory/inhibitory TMS over the left DLPFC (excitatory TMS resulting in higher liking and motivation, and vice versa for inhibitory TMS). The authors suggested that the key mechanism driving this effect was the modulation of fronto-striatal excitability driven by TMS over the left DLPFC. In particular, TMS may have modulated reward-anticipation mechanisms mediated by the dorsal striatum contributing to the aesthetic appreciation of music (Salimpoor et al. 2011). This resulted in an amplification (for excitatory TMS) or reduction (for inhibitory TMS) of the emotional response experienced by the listener, leading to the modulation of subjective liking.

What are the neural mechanisms mediating these effects? The findings reviewed in this section may be interpreted as reflecting indirect effects of prefrontal cortex stimulation on reward processing (and associated dopamine release) in subcortical structures (Chib et al. 2013). However, other mechanisms and networks may also be affected. Evidence for this comes from a combined tDCS-resting state fMRI study (Keeser et al. 2011) in which left DLPFC anodal tDCS modulated large-scale patterns of functional connectivity affecting both the default mode network and the fronto-parietal network, with both these networks being involved in aesthetic valuation (Cela-Conde et al. 2009, 2013; Vessel et al. 2013, 2019). The fronto-parietal network is believed to exert top-down attentional control directing attention toward task-relevant features (Waskom et al. 2014). Accordingly, activation in the left DLPFC during aesthetic viewing of paintings may reflect a top-down control in directing perception toward an aesthetic (vs. pragmatic) orientation, as suggested by Cupchik et al. (2009). Enhancing excitability of the left DLPFC may boost aesthetic orientation towards the viewed stimuli (Cattaneo et al. 2014b), whereas interfering with activity in this region may interfere with adoption of an aesthetic orientation resulting in decreased preference for viewed stimuli. This latter mechanism may account for the effects we reported in a prior study (Cattaneo et al. 2014a) where we interfered with left DLPFC (localized as F3 on the EEG cap) activity via TMS whilst observers valuated a series of paintings. Interestingly, we found that triple-pulse 10 Hz TMS over the left DLPFC selectively affected preference for participants’ art preferred category but not for the their other less preferred category (i.e., figurative or abstract, assessed prior to the experiment). We suggested that due to interference with top-down processing by left DLPFC TMS, cognitive resources might not have been allocated to the search and valuation of the esthetic qualities of the paintings that were generally liked more. Such an effect was not observed for the less generally liked sort of stimuli, possibly because participants oriented esthetically toward them to a lesser extent. This hypothesis still remains speculative at this stage, and deserves further testing. In fact, reward computation may also be particularly relevant for preferred art category and hence more prone to be modulated by stimulation; thus appropriate study design must be incorporated to disentangle these possibilities.

### Summary and discussion

The studies by Chib et al. (2013), Mas-Herrero et al. (2018), Cattaneo et al. (2014a, b) and Ferrari et al. (2015) suggest that the prefrontal cortex is causally involved in aesthetic appreciation. More than one mechanism may mediate this effect. Good candidates include the modulation of fronto-striatal excitability and of top-down attentional control by prefrontal cortex stimulation. Prefrontal stimulation may also directly affect the default mode network that has been found to activate during aesthetic experiences (Cela-Conde et al. 2013; Vessel et al. 2012, 2019). The observed hemispheric differences in the contribution of the DLPFC in aesthetic appreciation (directly assessed though only in case of face attractiveness, Chib et al. 2013; Ferrari et al. 2015) may depend on lateralized fronto-striatal connections but also on the specialization of the left and right prefrontal cortex for different types of processing (e.g., configural vs. feature-based). Admittedly, at this stage available brain stimulation results on the role of the DLPFC in aesthetic appreciation are still quite fragmentary, and are difficult to reconcile with a unique interpretation. In addition, the role of the medial PFC in aesthetic appreciation has only been little investigated (Chib et al. 2013; Nakamura and Kawabata 2015; see also Ferrari et al. 2017). Future studies should further investigate the functional significance of the contribution of the DLPFC and also of the mPFC to aesthetic experiences (note that the orbitofrontal cortex for its ventral position cannot be directly stimulated). This can be done by possibly combining brain stimulation with neuroimaging (as in Chib et al. 2013) as well as by varying the type of stimuli and task procedure used to selectively assess different mechanisms supported by prefrontal cortices that play a role in aesthetic valuation.

## Conclusions

Non-invasive brain stimulation can critically complement neuroimaging and electrophysiological evidence in research on neuroaesthetics by informing about brain-behavioral *causal* relationships. Overall, studies conducted so far seem to suggest that various regions along the visual dorsal and ventral pathways are involved in aesthetic valuation of bodies and artworks (Calvo-Merino et al. 2010; Cattaneo et al. 2015, 2017; Cazzato et al. 2014, 2016; Ferrari et al. 2015, 2018). Evidence on the role of motor and premotor cortices in contributing to aesthetic valuation appears to be more preliminary, with available TMS findings supporting a role of premotor cortices (Calvo-Merino et al. 2010; Cazzato et al. 2016) in mediating preference for bodies, whereas no brain stimulation study has directly assessed the contribution of premotor cortices to aesthetic valuation of artworks. MEP studies have reported modulation of cortico-spinal excitability induced by viewing paintings and dance (Battaglia et al. 2011; Jola and Grosbras 2013) but have not investigated whether preference for the viewed stimuli affect MEP amplitude. TMS and tDCS studies have also provided support for a causal involvement of the dorsolateral prefrontal cortex in visual aesthetic decisions (Cattaneo et al. 2014a, b; Chib et al. 2013; Ferrari et al. 2015; see also Mas-Herrero et al. 2018 for music aesthetic appreciation), but the mechanisms mediating this effect (e.g., modulation of fronto-striatal connectivity, interference with top-down attentional control) are still yet to be clarified.

Overall, the studies discussed in this review highly differed both in terms of stimulation parameters and for the type of task and the stimuli used, making it hard (at least at this stage) to draw strong conclusions about the possible causal contribution of different cortical regions in aesthetic appreciation. Moreover, as is often the case in studies employing non-invasive brain stimulation (for a critical discussion, see for instance Héroux et al. 2015) as well as in research in neuroscience in general (e.g., Button et al. 2013), the studies reviewed here have relied on relatively small sample sizes, especially when considering the high intra- and inter-individual variability in TMS and tDCS responses (for recent evidence, see Filmer et al. 2019; Iyer et al. 2019; Lee et al. 2018; Rocchi et al. 2018). In fact, the number of participants for an experimental group (i.e., groups based on individual differences or assigned to different task conditions) in the TMS and tDCS studies considered in this review have ranged from 7 to 20. Given this methodological limitation related to low statistical power, the risk that at least some of the reviewed significant findings were indeed false positive findings is particularly high, also considering that none of the reviewed works provided a replication of significant effects within the same study. In some cases, the same group used a similar approach and similar stimuli in different studies (for instance, Cazzato et al. (2014) and (2016) studies), but results were not entirely consistent. Moreover, the results of studies by different groups targeting the same region (even when considering differences in experimental paradigms and stimuli employed) led to inconsistent results overall (see for instance the different effects of anodal stimulation of the right DLPFC on aesthetic appreciation of faces reported by Chib et al. 2013, and by Ferrari et al. 2015). These limitations notwithstanding, the potential contribution of non-invasive brain stimulation to research in neuroaesthetics remains important and continued effort should be made to replicate available findings in larger samples, so as to provide a more solid foundation for future experiments.

Several aspects remain to be investigated, such as whether and how the context, culture and an individual’s expertise with the viewed stimuli (the *meaning*-*knowledge* system) affect the recruitment of different brain regions during aesthetic appreciation (see for instance Yang et al. 2019, for a recent fMRI study on how culture may shape brain responses to aesthetic evaluation of Eastern and Western landscapes). Hemispheric differences in the effects of TMS over both sensory and prefrontal regions during aesthetic valuation observed in some of the reviewed studies (Cazzato et al. 2014; Chib et al. 2013; Ferrari et al. 2015) also deserve further investigation. Moreover, chronometric TMS approaches (i.e., TMS delivered at different time-points from stimulus onset, for a review see de Graaf et al. 2014) would help shed light on the time-course of aesthetic valuation in different cortical regions. Another intriguing approach would be to use frequency-tuned stimulation to interact with endogenous oscillatory neural activity during aesthetic engagement. Rhythmic TMS and alternate current stimulation (tACS, a form of transcranial electrical stimulation) have been increasingly used in cognitive neuroscience to modulate brain oscillations in a frequency specific manner to drive network activity (and associated functions) by entraining ongoing brain oscillations or synchronizing neuronal networks (Romei et al. 2016). This approach has already led to important developments in the understanding of the neural bases of cognitive functions (Albouy et al. 2018). If reward is associated to specific oscillatory neural activity (Levy et al. 2017; Marco-Pallarés et al. 2015), brain stimulation may be used to boost oscillations associated with art-related appreciation (reward). Finally, combining non-invasive brain stimulation with fMRI (Chib et al. 2013) or EEG recording will enable a better understanding of the interrelation between the physiological and behavioral impact of brain stimulation on aesthetic valuation. The combined use of these methods represents a growing trend in cognitive neuroscience (Farzan et al. 2016; Saiote et al. 2013), and would greatly benefit research in neuroaesthetics.

## Footnotes

1 Whereas only a few studies have tested the effect of focal brain damage on aesthetic appreciation, in recent years there has been an increasing interest in investigating the impact of neurodegenerative disorders on the appreciation of visual art (e.g., Boutoleau-Bretonnière et al. 2016; Graham et al. 2013; Halpern et al. 2008). Moreover, note that this review does not consider art creation/production, a field in which the effects of focal brain damage have been largely investigated (e.g., Chatterjee 2011).

2 Studies considered in this review were selected using a Pubmed search and the terms “aesthetic/esthetic”, “beauty”, “attractiveness”, “liking”, “pleasure”, “artworks”, “paintings”, “faces”, “preference”, “bodies” combined with (< AND >) “brain stimulation”, “TMS”, “tDCS”, “tACS”, “tRNS”, “tES” (last updated in July 2019). I then selected only brain-stimulation studies specifically dealing with visual aesthetic appreciation and checked whether they referred to other relevant studies possibly not indexed in Pubmed. Following this search criteria, 13 studies were selected (6 of which were authored by me). The relatively low number of brain stimulation studies available reflect the fact that neuroaesthetics is still a relatively “young” field compared to other areas of research in cognitive neuroscience.
